# The Impact of Afforestation on Soil Organic Carbon Sequestration on the Qinghai Plateau, China

**DOI:** 10.1371/journal.pone.0116591

**Published:** 2015-02-23

**Authors:** Sheng-wei Shi, Peng-fei Han, Ping Zhang, Fan Ding, Cheng-lin Ma

**Affiliations:** 1 State Key Laboratory of Atmospheric Boundary Layer Physics and Atmospheric Chemistry, Institute of Atmospheric Physics, Chinese Academy of Sciences, Beijing, 100083, PR China; 2 University of Chinese Academy of Sciences, Beijing 100049, PR China; 3 College of Forestry, Northwest A&F University, Yangling, Shaanxi 712100, PR China; 4 State Key Laboratory of Vegetation and Environmental Change, Institute of Botany, Chinese Academy of Sciences, Beijing 100093, PR China; Fudan University, CHINA

## Abstract

Afforestation, the conversion of non-forested land into forest, is widespread in China. However, the dynamics of soil organic carbon (SOC) after afforestation are not well understood, especially in plateau climate zones. For a total of 48 shrub- and/or tree-dominated afforestation sites on the Qinghai Plateau, Northwestern China, post-afforestation changes in SOC, total nitrogen (TN), the carbon-to-nitrogen ratio (C/N) and soil bulk density (BD) were investigated to a soil depth of 60 cm using the paired-plots method. SOC and TN accumulated at rates of 138.2 g C m^-2^ yr^-1^ and 4.6 g N m^-2^ yr^-1^, respectively, in shrub-dominated afforestation sites and at rates of 113.3 g C m^-2^ yr-^-1^ and 6.7 g N m^-2^yr^-1^, respectively, in tree-dominated afforestation sites. Soil BD was slightly reduced in all layers in the shrub-dominated afforestation plots, and significantly reduced in soil layers from 0–40cm in the tree-dominated afforestation plots. The C/N ratio was higher in afforested sites relative to the reference sites. SOC accumulation was closely related to TN accumulation following afforestation, and the inclusion of N-fixing species in tree-dominated afforestation sites additionally increased the soil accumulation capacity for SOC (*p* < 0.05). Multiple regression models including the age of an afforestation plot and total number of plant species explained 75% of the variation in relative SOC content change at depth of 0–20 cm, in tree-dominated afforestation sites. We conclude that afforestation on the Qinghai Plateau is associated with great capability of SOC and TN sequestration. This study improves our understanding of the mechanisms underlying SOC and TN accumulation in a plateau climate, and provides evidence on the C sequestration potentials associated with forestry projects in China.

## Introduction

Soil is a major carbon (C) pool in terrestrial ecosystems, containing nearly 1500 Pg of C as soil organic carbon (SOC) in the first meter of depth [1]. The dynamics of SOC, which is prone to loss or gain due to land-use changes [2], are critical to understand, owing to the increasing carbon dioxide (CO_2_) concentration in the atmosphere [3]. Losses of soil C caused by the cultivation of grassland and by deforestation are the second greatest source of anthropogenic greenhouse gas emissions [3,4]. Land C emissions contributed about 36% of the anthropogenic CO_2_ emitted into the atmosphere from 1985–2000 [4]. Afforestation, the conversion of non-forested land into forest, is one of the cost-effective strategies for climate change mitigation, owing to the ability of forested land to sequester CO_2_ from the atmosphere, storing it in woody biomass via plant photosynthesis and soil organic matter via humification [5,6]. Afforestation also protects soils against wind and water erosion [7,8], and increases soil C stability by forming macroaggregates through mycorrhizal associations with plant roots and soil microbes [9,10]. However, both the magnitude and direction of soil C dynamics following afforestation are poorly characterized in the literature, with different studies sometimes showing inconsistent results. For example, the SOC stock in the top 10 cm of soils was enhanced by only 20% after afforestation of cropland in Northern Europe [11], but increased by 68.6% in the top 20 cm of soils in China [12]. Previous reviews of this issue also report that there is a high risk of soil C depletion in young stands established on cropland [13,14], and in forests established on grassland [2,11,15]. Additionally, changes in SOC following afforestation are directly related to the prior land use, environmental conditions (climatic factors, plant species composition and intrinsic edaphic properties) and human management [16–19]. Thus, a credible assessment of SOC sequestration following afforestation at regional scale remains a challenge, owing to the need for such comprehensive information [20].

Nitrogen is a constituent of soil organic matter (SOM) that directly influences SOC accumulation via its influence on the input rate from net primary productivity (NPP). Hence, soil N can be an important factor in the regulation of long-term C sequestration potential in terrestrial ecosystems [21–23]. N-fixing plant species can substantially add to the amount of available N in the soil via biological N-fixation [19,24]. This increase in N can decrease microbial respiration rates [25,26], thus facilitate C sequestration and improve soil fertility in forested land. The amount of C sequestered in soils following afforestation is directly related to levels of N retention; for example, in a planted forest, a gain of 1 g total soil N (TN) was accompanied by a 35 g and a 7 g gain of C in the O horizon and the mineral soil layers (in the first meter of depth), respectively [18]. Although there have been many studies of N effects on terrestrial C cycles and the underlying mechanisms, whether or not the SOC sequestration potentials of afforested lands will be restricted in the long term by progressive N limitation remains controversial [22,27,28]. Hence, it is crucial not only to follow standardized protocols when collecting post-afforestation data on SOC dynamics, but also to include measurements of soil N dynamics in field experiments, in order to credibly assess SOC sequestration potentials and mechanisms [29].

The Qinghai-Tibet Plateau, which covers nearly one-fourth of China’s territory, is the world’s highest plateau and represents a unique plateau climate [30]. Gaining an understanding of post-afforestation changes in SOC and TN within this climatic zone is critical for two key reasons. Firstly, it is well-known that the response of SOC to afforestation can vary significantly across geographic and/or climatic zones [11,12,17,18]. At a global scale, studies of SOC sequestration potentials are geographically biased tropical and temperate climatic zones [16,18,31,32], with few studies examining post-afforestation SOC dynamics in the plateau climatic zone [33,34]. This bias limits our understanding of the mechanisms underlying SOC changes at a global scale and makes it difficult to reconcile divergent results from different climate zones. Secondly, the responses of both SOC and TN to afforestation have been shown to vary significantly with the use of different plant species and types of afforestation (afforestation of cropland or barren land, mixed forests or pure forests) [17,24]. However, the majority of reviews published to date examining the influence of tree species planted on SOC and TN stocks have looked across diverse climatic zones [17,18], with little research within similar zones.

An opportunity to examine the impact of planted tree species within a single climatic zone exists on the Qinghai-Tibet Plateau, where a large planted forest was established using multiple species. In Qinghai province, shrubland now occupies up to 52.9% of the total planted area [35]. Study of this unique multi-species forest could illuminate the individual responses of SOC and TN, as well as their interaction, to afforestation (using different tree species) in a unique plateau climatic zone. However, this opportunity has yet to be fully exploited, with little research occurring to date, particularly for shrubland. The scientific identification of an afforestation type that combines the desire for C sequestration with the maintence of soil fertility is essential for forestry policy-makers. Consequently, it is a priority to evaluate changes in SOC accurately within this unique climatic region.

In the current study, we examined changes in SOC and total soil nitrogen (TN) following afforestation on the Qinghai-Tibet Plateau, a region for which little is known about C dynamics. We examined 48 sites, using the same protocol to investigate the dynamics of SOC, TN, carbon-to-nitrogen ratio (C/N) and soil bulk density (BD) after afforestation in this region. Our objectives were to: (1) evaluate the direction and magnitude of changes in SOC and TN stocks after afforestation across the Qinghai Plateau using the paired-plot method; and (2) understand the mechanisms underlying the SOC and TN accumulation observed after afforestation of cropland on the Qinghai Plateau.

## Materials and Methods

### Site Descriptions

Soil sample collection sites were selected on the Qinghai Plateau in accordance with the distribution of forest types and corresponding afforested areas; sampling was permitted by the Chinese Academy of Sciences and the Forestry Department of Qinghai Province. According to the Forestry Statistic Yearbook for Qinghai province, five prefectures (Haibei, Haidong, Hainan, Haixi, and Xining) in the north of Qinghai province account for 97% of the province’s afforested areas. Although the southern part of the province (consisting of Huangnan, Guoluo, and Yushu prefectures) accounts for a high proportion (40%) of the total land area, it accounts for only 4% of the total area afforested from 2002–2010 [35]. Thus, the sample sites were focused in northern Qinghai province and were categorized according to forest type and proportion of the land that was afforested in the last decades.

**Fig 1:**
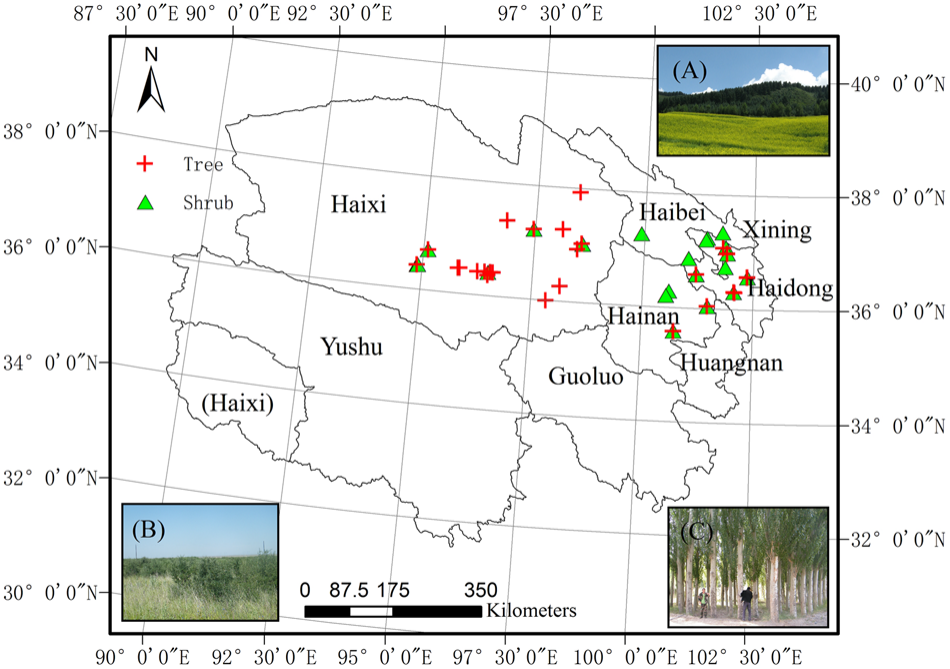
Location of the 48 sample sites in Qinghai province. The three plates in the corners of the map show the typical forest types. (A) A 21-year-old planted forest (*Larch*) under rain-fed conditions in the eastern hilly region, (B) a 9-year-old planted shrubland (*Hippophae rhamnoides*) in the middle region, and (C) a 31-year-old planted forest (*Populus bolleana*) with irrigation in the west region.

In order to investigate changes in SOC and TN correlated with afforestation, each afforested sample site plot was paired with a nearby non-afforested (reference) plot, either cropland without organic fertilizer inputs or barren land. To ensure that paired sample plots were comparable, prospective study sites had to meet the following criteria: (1) afforested land had been under cultivation for at least 20 years prior to afforestation, to ensure that any differences in SOC were due to afforestation itself. As little soil C is accumulated in cropland, this was assumed to be a long enough period that observed soil C stock would be the result of recent afforestation [31]; (2) the plantation age of afforested land was recorded accurately; (3) the paired plots had comparable soil types with similar topographies; and, (4) the distance between paired plots was no greater than 1,000 meters. The age and land use history of the afforested plots were acquired from local county officials or town forestry administration bureaus or stations. A total of 48 afforested sites were included in the study (Fig. 1): 24 shrubland sites, 20 broadleaf forest sites and 4 conifer-dominated sites. The land used for soil core sampling was protected by the Forestry Department of Qinghai province. Sites located in the hills on the eastern Qinghai Plateau had relatively longer distances (400~1,000 m) between paired plots than sites located in central and western Qinghai Plateau (within 100~400 m). Afforestation plots were classified as either shrub-dominated (24 sites) or tree-dominated (24 sites), based on the life form of the dominant plant species. The shrub-dominated plots were further subdivided into shrub-grass ecosystems (12 sites) and pure shrub plantations (12 sites) according to the mode of afforestation. The tree-dominated plots were subdivided into mixed-forests with N-fixers (13 sites) and forests without N-fixers (11 sites), based on the N fixation function of accompanying species. The land-use types that existed prior to afforestation were also subdivided into cropland (42 sites) and barren land (6 sites). This research did not involve endangered or protected species. Additionally, this study did not involve animal husbandry, experimentation, or care/welfare. Basic information on each sample site (including climatic factors, plant species and GPS coordinates) is provided in S1 Table.

### Soil Sampling and Analysis

**Fig 2:**
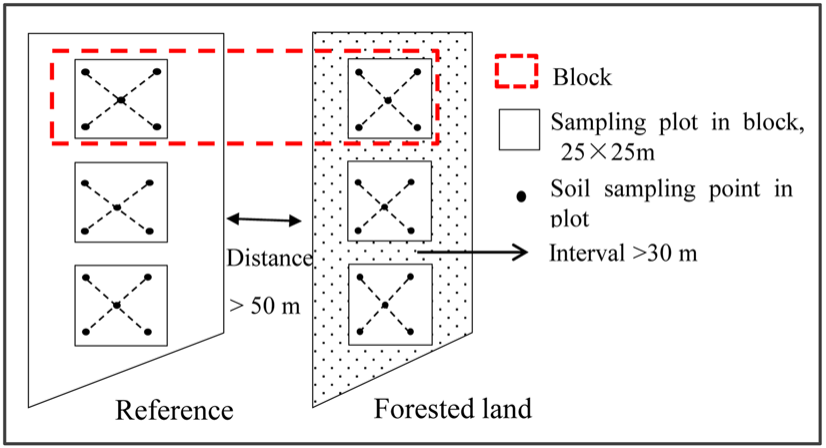
Scale map of soil sampling locations at one site on the Qinghai Plateau.

Field sampling took place between July and October 2011. The same sampling protocol was used for all sites (Fig. 2). The paired plots covered at least 25×25 m^2^ each, with 3 replicates per sample site. All soil cores were taken using a stainless steel auger (3.5 cm in diameter) with fixed intervals of 0–10, 10–20, 20–40, 40–60, 60–80 and 80–100 cm in the mineral soil layer. To account for spatial heterogeneity within each sampling plot, five soil cores were obtained for each interval in each plot; these were mixed in a cotton bag. An obvious organic layer (forest floor) was noted in older afforested plots of a few decades of age; a sample of this layer was collected from a 20×20 cm square. However, there was no clear evidence of the presence of forest floor (< 1 cm) in most of the forested plots, especially for shrub-dominated sites and younger tree-dominated sites (< 15 years old) (S1 Table). Thus, only four sites with relatively older ages, including two sites with 26- and 29-year *Larix principis-rupprechtii* forest, a 33-year *Populus cathayana Rehd*. forest and a 57-year *Populus cathayana Lauche* forest, were selected to measure the C and N accumulation in the forest floor. Many of the soil profiles obtained in the sampling plots had a depth of less than 1 m. Therefore, we only studied the changes in SOC and TN that occurred from 0–60 cm, and the soil cores deeper than 60 cm were used to correct the changes in SOC and TN stocks via the equivalent mass method [31,36]. All of the soil samples were air-dried in a well-ventilated room. Each soil sample was sieved through a 2 mm mesh, and the remaining fraction, > 2 mm, was separated and weighed. Soil bulk density samples were also collected at a depth of 0–60 cm, with 10 cm intervals, using stainless steel bulk density rings (AMS soil sampling equipment, USA) with three replicates per afforested sites and reference plot. The soil bulk density samples were dried in an oven in the laboratory at 105°C until reaching constant in weight. The dominant and accompanying species were recorded for each sample site.

The soil samples < 2 mm were ground finely using a ball mill after removing any fine roots and then passed through a 0.25 mm mesh for measurement of SOC and TN concentrations. The SOC concentration was determined using the K_2_Cr_2_O_7_–H_2_SO_4_ wet oxidation method of Walkley and Black [37]. The TN concentration was analyzed with a continuous-flow auto-analyzer (Auto-Analyzer III, Bran+Luebbe GmbH, Germany) after the samples were digested with concentrated H_2_SO_4_ (98%). The C/N was calculated from the SOC and TN concentrations. The soil texture, clay (< 0.002 mm), silt (0.002–0.05 mm), and sand (0.05–2 mm) content in the soil samples were determined using a Laser Particle Size Analyzer.

### Main Calculations

All data for the soil samples, such as sample locations, soil properties and plant types, was compiled using Microsoft Excel. The SOC content in a fixed layer, *i*, was calculated using [38]:

$$C_{i}=SOC_{i}\times BD_{i}\times h_{i}\times (1-\delta_{i})\times 10^{-1}$$ (1)

$$C_{t}=\sum_{i=1}^{n}C_{i}$$ (2)

where *C*_*i*_ is the SOC content (Mg C ha^-1^) and *SOC*_*i*_ is the SOC concentration (g C kg^-1^) in sample layer *i*; *BD*_*i*_ is the bulk density (g cm^-3^); *h*_*i*_ is the thickness (cm) of the soil layer; *δ*_*i*_ is the fraction (%) of coarse fraction > 2 mm; and *C*_*t*_ is the SOC stock in the soil profile, which is the sum of the content in different layers.

The equivalent mass method was used to evaluate the changes in SOC stocks after afforestation due to soil BD changes. The SOC stock value obtained after afforestation was corrected to the same mass as in the reference site using the equations reported by Poeplau et al. [31].

Calculation of the change in SOC stocks (Δ*C*_*j*_, Mg C ha^-1^) for sample site *j* followed equation:

$$\Delta C_{j}=C_{ej}-C_{cj}$$ (3)

where *C*_*ej*_ is the SOC stock for sample site *j* for afforested soil (Mg C ha^-1^), and *C*_*cj*_ is the SOC stock in the reference (Mg C ha^-1^). The relative change in SOC stock after afforestation (*P*_*j*_, %) for sample site *j* was estimated as [17]:

$$P_{j}=\frac{\Delta C_{j}}{C_{cj}}\times 100$$ (4)

The rate of absolute changes in SOC stocks (${\overset{-}{R}}_{j}$, g C m^-2^ yr^-1^) and the rate of relative SOC stock change (${\overset{-}{p}}_{j}$, % yr^-1^) were calculated, to evaluate the changes in SOC intensity, using the formula described by Li et al. [18].

The TN contents and intensity of changes in TN stocks were also calculated using corresponding forms of equations (1) through (4).

### Statistical Analysis

There was substantial variation among the soil properties in their variance at each sample site, indicating that not all the data had the same quality. Therefore, we decided to use weighted regression models to explore which factors most strongly influenced the dynamics of SOC and TN stocks after afforestation. We calculated the mean and standard deviation of each soil property measured for each paired afforested and reference plot. Next, the property variances were estimated using the equation [39]:

$$\sigma_{j}^{2}=\frac{S_{ej}^{2}}{n_{ej}X_{ej}^{2}}+\frac{S_{cj}^{2}}{n_{cj}X_{cj}^{2}}$$ (5)

where $\sigma_{j}^{2}$ is the variance of a response variables (i.e. soil property) after afforestation at site *j*; *X*_ej_ is the mean of the variable for the afforested plots and *X*_cj_ is the mean for reference plots at site *j*; *n*_*ej*_ and *n*_*cj*_ are numbers of samples obtained in the paired afforested and reference plots, respectively; and *S*_*ej*_ and *S*_*cj*_ are the standard deviations for the paired afforested and reference plots, respectively.

In order to analyze the effects of different potential causal factors on the relative changes in SOC and TN stocks, the C/N ratio and soil BD associated with afforestation, forward/backward regressions were performed using the inverse variance ($1/\sigma_{j}^{2}$) as weighted factor [40,41].

We calculated the annual availability of water, *W*, for use as an index of climatic effects in modeling the response of SOC and TN stocks to afforestation, using the equation [42,43]:

$$W=MAP-\frac{Q_{s}}{\rho_{w}L}+4000$$ (6)

where MAP is mean annual precipitation (mm yr^-1^), Q_s_ is mean annual global solar radiation (J m^2^ yr^-1^), *ρ*_*w*_ is density of liquid water (1000 kg m^-3^ at 25°C) and L is the latent heat of water evaporation (2.5×10^6^ J/ kg H_2_O at 25°C).

We compared how SOC and TN stocks changed with afforestation (i.e. comparing reference vs. afforested plots) using a mixed model with spatial autocorrelation, over soil depth, in SAS (Sas Institute Inc., 1999). The Kenward-Roger correction was applied to the denominator to correct the degrees of freedom, as in the statistical method reported by Eclesia et al. [44]. A mixed model, with sample site as a random factor, was used to test for differences in the rate of SOC or TN content in each soil layer among the different afforestation types. Linear regression was used to explore the association between SOC and TN change intensity in the top 20 cm for each afforestation type.

## Results

### Rates of Change in SOC and TN Content

Among the shrub-dominated afforestation plots, SOC content in the shrub-grass ecosystems increased significantly at a rate of 50.5 g C m^-2^ yr^-1^ in surface soils with a depth of 0–10 cm (*p* < 0.05) (Table 1). The SOC content in layers deeper than 10 cm and the TN content in each soil layer increased slightly but not significantly (i.e. *p* > 0.05). In contrast, SOC content in pure shrub plantations increased significantly in the deeper soil layers, but not in the top 10 cm (*p* < 0.05). Differences in the rates of change in SOC and TN contents between the shrub-grass ecosystems and pure shrub plantations were not significant, except for TN content at 0–10 cm (*p* = 0.03). Overall, the mean rates of change in SOC and TN contents within the top 60 cm of the soil were 138.2 g C m^-2^ yr^-1^ and 4.6 g N m^-2^ yr^-1^, respectively.

**Table 1:** SOC and TN contents and their changes after afforestation with shrubs and trees on the Qinghai Plateau.^a^

	SOC					TN				
Soil depths	Afforestation	Control	ΔSOC	C.V. ^b^	R_SOC_	Afforestation	Control	ΔTN	C.V.	R_TN_
(cm)	(Mg C ha^-1^)	(Mg C ha^-1^)	(Mg C ha^-1^)	(%)	(g C m^-2^ yr^-1^)	(Mg N ha^-1^)	(Mg N ha^-1^)	(Mg N ha^-1^)	(%)	(g N m^-2^ yr^-1^)
Shrub-dominated afforestation plots (shrub-grass ecosystems)										
0–10	20.21	14.43	5.78*	143	50.46*	2.02	1.77	0.24	82	2.12*
10–20	16.87	13.87	3.02	158	26.32^†^	2.08	1.89	0.19	199	1.62
20–40	29.58	25.73	3.85	249	33.62	3.43	3.57	-0.13	-496	-1.15
40–60	24.91	22.95	1.95	451	17.05	3.2	3.04	0.16	737	1.35
Shrub-dominated afforestation plots (pure shrub plantations)										
0–10	17.71	14.63	3.09	218	27.44^†^	1.77	1.66	0.11	653	1.04
10–20	15.55	11.21	4.34^†^	116	38.58*	1.76	1.66	0.1	419	0.85
20–40	24.47	20.03	4.44^†^	181	39.47	3.06	2.88	0.18	415	1.64
40–60	22.53	17.95	4.58^†^	129	40.73*	2.93	2.75	0.18^†^	596	1.64
Tree-dominated afforestation plots (mixed forests with N-fixers)										
0–10	18.57	11.18	7.39**	127	39.89**	1.76	1.32	0.43^†^	112	2.33**
10–20	14.59	10.36	4.23	182	22.82	1.56	1.24	0.32	145	1.71*
20–40	23.58	17.88	5.70^†^	190	28.67	2.74	2.31	0.43^†^	318	2.34
40–60	20.95	17.24	3.71	215	20.04	2.57	2.37	0.2	214	1.09
Tree-dominated afforestation plots (forests without N-fixers)										
0–10	15.8	9.86	5.94*	142	33.18*	1.66	1.47	0.19	322	1.07
10–20	12.97	10.2	2.77	198	15.46	1.57	1.55	0.02	1824	0.12
20–40	25.88	19.36	6.51**	80	36.40**	3.46	2.94	0.52^†^	232	2.89^†^
40–60	22.47	19.54	2.93	188	16.39	2.93	2.6	0.33	194	1.87

^a^ The different units can be converted using a transfer coefficient (e.g., 1 Mg ha^-1^ = 100 g m^-2^).

^b^ C.V.: coefficient of variation for changes in SOC and TN content after afforestation.

^†^ *p* < 0.1;

* *p* < 0.05;

** *p* < 0.01, tested with a mixed model.

Among the tree-dominated afforestation plots, the SOC content in mixed forests including N-fixing species increased significantly by 7.4 Mg C ha^-1^ at 0–10 cm of soil depth (*p* < 0.01) and by 5.7 Mg C ha^-1^ at 20–40 cm of depth (*p* < 0. 1) (Table 1). The TN content increased significantly at a rate of 2.3 g N m^-2^ yr^-1^ at 0–10 cm (*p* < 0.01) and by 1.7 g N m^-2^ yr^-1^ at 10–20 cm (*p* < 0.05). Similarly, the SOC content in forests without N-fixing species increased significantly (*p* < 0.05), at a rate of 33.2 g C m^-2^ yr^-1^ at 0–10 cm and 36.4 g C m^-2^ yr^-1^ at 20–40 cm. Overall, the mean rate of change in SOC and TN contents within the top 60 cm were 113.3 g C m^-2^ yr^-1^ and 6.7 g N m^-2^ yr^-1^, respectively. Rates of change in SOC and TN contents in the top 10 cm of the soil differed significantly (*p* < 0.05) between forests with and without N-fixing species.

**Fig 3:**
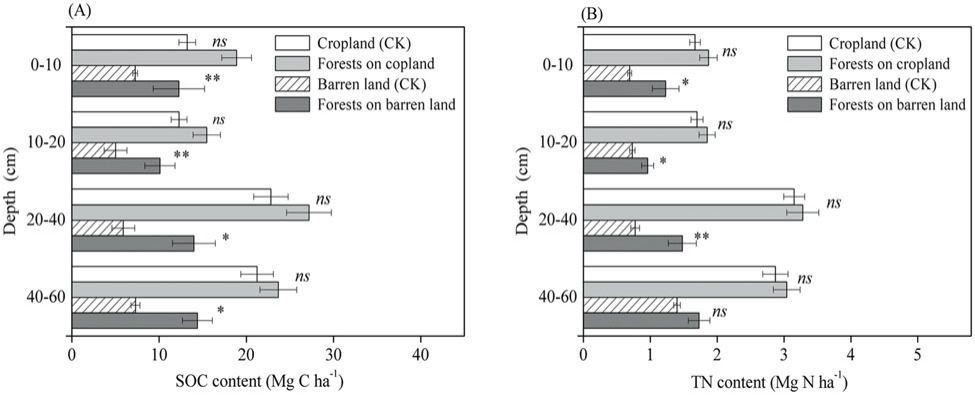
Comparing the mean soil SOC (A) and soil TN (B) contents in afforested and reference (CK) plots at different soil layers across multiple sites on the Qinghai Plateau. The symbols of **, * and *ns* indicate the levels of significant differences between reference and afforested plots at <0.01, <0.05 and >0.05, respectively.

Prior land use of the afforested plots had limited effects on post-afforestation soil changes. The SOC content in each soil layer was significantly greater (*p* < 0.05) in both types of afforested plots than in the reference plots, with the exception of afforestation plots in former cropland at 0–10 cm (*p* > 0.05) (Fig. 3A). The TN content in each soil layer at the afforestation plots in former cropland increased slightly but not significantly (Fig. 3B). However, the TN content in afforestation plots in former barren land increased significantly at all soil intervals within 0–40 cm (*p* < 0.05). Notably, the forest floor in the oldest afforested plots (several decades old) sequestered substantial amounts of C and N after afforestation with either conifers or deciduous trees, although this result is based on a limited number of sites (S1 Fig.).

### Patterns in the Rate of Change of SOC and TN Content

**Fig 4:**
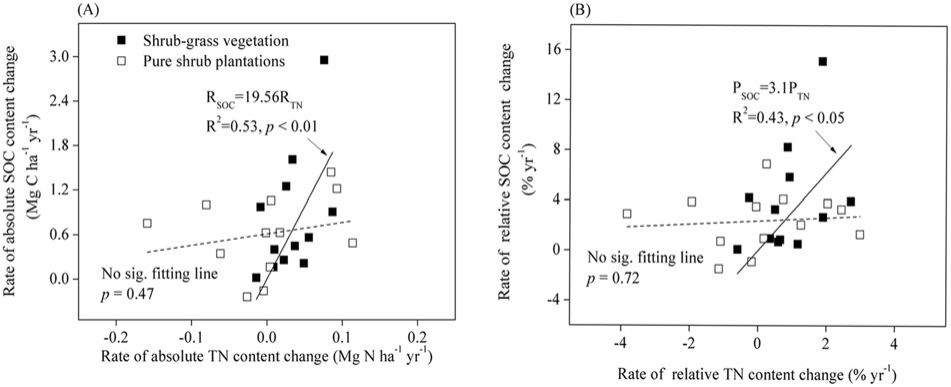
Patterns in SOC and TN content at 0–20 cm in shrub-dominated afforested sites. (A) Rates of absolute change in SOC and TN content; (B) Rates of relative change in SOC and TN content. The solid line indicates shrub-grass ecosystems and the dashed line indicates pure shrub plantations.

**Fig 5:**
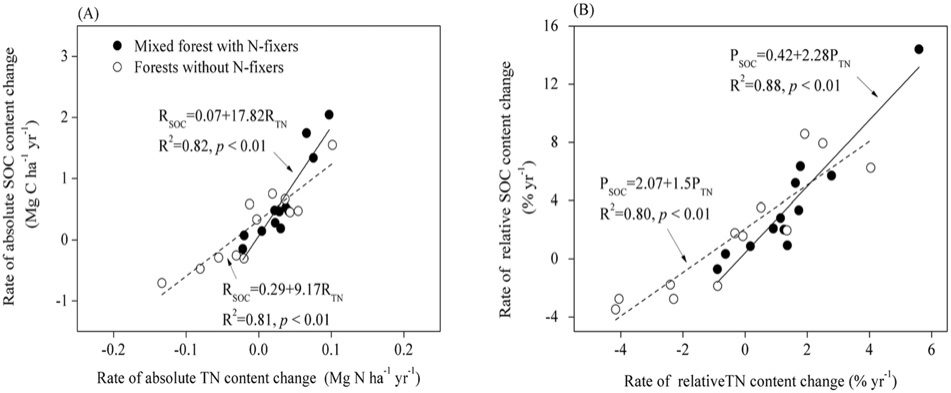
Patterns in SOC and TN content at 0–20 cm in tree-dominated afforestation systems. (A) Rates of absolute change in SOC and TN content; (B) Rates of relative change in SOC and TN content. The solid line indicates mixed forests with N-fixers and the dashed line indicates forests without N-fixers.

In the shrub-dominated afforestation plots, there was a weak overall relationship between the rate of change of SOC content and that of TN content, but no significant relationship in pure shrub plantations (Fig. 4A, B). The slope of the regression between these two rates of change was 3.1 (Fig. 4B), which was greater than 1 (*p* < 0.01). In the tree-dominated afforestation plots, there was a strong linear relationship between the rate of change of SOC content and that of TN content after afforestation (Fig. 5). Here, a 1.0 Mg gain in TN was accompanied by a 17.8 Mg SOC gain in forests with N-fixers and by a 9.2 Mg C gain in forests without N-fixers (Fig. 5A). Statistical analysis indicated that the slopes of the regressions of relative change in SOC content vs. relative change in TN content obtained for forest with N-fixers (2.3) and without N-fixers (1.5) were both greater than 1 and differed significantly from each other (*p* < 0.01) (Fig. 5B).

### Changes in Soil BD and the C/N Ratio

**Fig 6:**
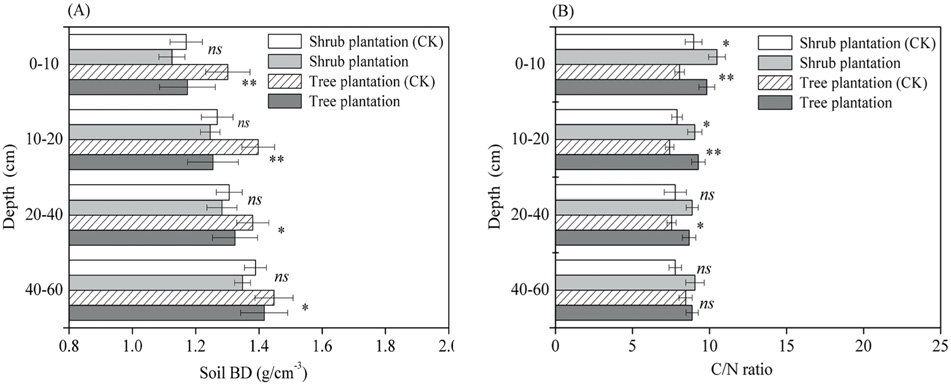
Comparing the mean soil BD (A) and C/N ratio (B) in afforested and reference (CK) plots at different soil layers across multiple sites on the Qinghai Plateau. The symbols of **, * and *ns* represent the levels of significant differences between reference and forested plots at < 0.01, < 0.05 and > 0.05, respectively.

Soil BD was only slightly reduced in all soil layers in the shrub-dominated afforestation plots (*p* > 0.05), while it was significantly reduced in the tree-dominated afforestation plots (Fig. 6A). The C/N ratio was significantly higher in afforested versus reference plots at soil depths of less than 20 cm,, increasing by 16.9% in the 0–10 cm soil depth interval and by 14.5% in the 10–20 cm interval (*p* < 0.05) (Fig. 6B). The C/N ratio in the tree-dominated afforestation plots was also significantly higher than in the reference plots at soil depths less than 40 cm (*p* < 0.05) (Fig. 6B).

### Determinants of SOC and TN Stock Changes after Afforestation

The relative change in SOC content with afforestation, for both shrub- and tree-dominated sites, was positively correlated with the age of the afforested site (Table 2). Examining the relevant climatic factors, in shrub-dominated afforested sites, the relative change in SOC content in the top 20 cm of soil was positively correlated with the mean annual temperature (*r* = 0.36, *p* < 0.1), and negatively correlated with the annual availability of water (*r* = -0.42, *p* < 0.1). In contrast, the mean annual temperature and annual availability of water had no significant relationship with SOC content change in tree-dominated afforested sites. The relative change in SOC content in the top 20 cm of soil in tree-dominated afforested sites was closely correlated with the total number of plant species (*r* = 0.62, *p* < 0.001). A multiple regression model containing afforestation age and the total number of plant species explained 75% of the relative SOC content changes in tree-dominated afforested sites (Table 2). However, in shrub-dominated afforested sites, the responses of SOC and TN to afforestation were poorly characterized by these factors (*R* ^2^ = 0.33 and 0.26, respectively).

**Table 2:** Multiple regression models for each response variable in each afforested system was established on the plantation age (Age), mean annual temperature (MAT), mean annual availability water (W) and the number of plant species (S).

Afforestation modes	Response variable	Regression parameters for response variable of each afforested system						
		Intercept	Age	MAT	W	S	Overall *R* ^*2*^	Overall *F*
Shrub-dominated afforestation system(0–20cm)	SOC (%)	73.31^†^	1.83^†^	4.61^†^	-0.04^†^	*ns*	0.33	3.46*
	TN (%)	80.43***	Ns	-3.69*	-0.03*	*ns*	0.26	0.94*
	C/N (%)	*ns*	*Ns*	*Ns*	*ns*	*ns*		
	BD (%)	*22*.*30* *	*Ns*	*Ns*	*-0*.*014* *	*ns*	0.21	6.13*
Tree-dominated afforestation system(0–20cm)	SOC (%)	-59.49***	4.863***	*Ns*	*ns*	19.59***	0.75	43.65***
	TN (%)	-23.22*	2.16***	*Ns*	-0.05*	9.17*	0.44	7.58***
	C/N (%)	13.95^†^	0.95^†^	*Ns*	*ns*	*ns*	0.11	3.52^†^
	BD (%)	7.29*	-0.56**	*Ns*	*ns*	*-3*.*13* *	0.42	10.65***

The coefficients in the final models which only included the effects of the significant level at *p* < 0.1.

^†^ *p* < 0.1;

* *p* < 0.05;

** *p* < 0.01;

*** *p* < 0.001 and *ns*, not significant.

## Discussion

### Effect of Afforestation on SOC Sequestration

The impacts of afforestation on SOC sequestration is inconsistent across published studies, varying from significant depletion [45,46], to negligible change [47] to dramatic increase of up to 163 g C m^-2^ yr^-1^ [48,49]. The average rate of SOC accumulation globally (at soil depths < 30 cm), following 19 years of afforestation on former grasslands and croplands, was 14.1 g C m^-2^ yr^-1^ [13], while the average rate was 36.7 g C m^-2^ yr^-1^ (soil depths < 20 cm) after 15 years of restoration of cropland in China [14]. According to a recent global meta-analysis [18], the rate of SOC accumulation in the top meter of soil, after conversion of cropland into forest, averaged 96.7 g C m^-2^ yr^-1^. Wang et al. [49], in a study of 159 afforested plots in Northeastern China, found that the rate of SOC accumulation after conversion of cropland into larch (*Larix gmelinii*) plantations averaged 96.4 g C m^-2^ yr^-1^, ranging from 57.9–139.4 g C m^-2^ yr^-1^, at soil depths of 0–20 cm. In this study, on the Qinghai Plateau, SOC accumulation rates (in soil depths of up to 60 cm) increased substantially with afforestation at a rate of 138.2 g C m^-2^ yr^-1^ and 113.3 g C m^-2^ yr^-1^, respectively, in shrub- and tree-based afforested plots (Table 1), indicating that afforestation is associated with greater SOC sequestration capability.

The magnitude of C accumulation in soils remains a poorly characterized aspect of the terrestrial C cycle [50]. As outlined previously in the literature, soil accounted for 30% of the total C sink (biomass and soil) in European forest ecosystems [51], and for 25–49% in United States forest ecosystems [52]. Net primary production (NPP), i.e. gross primary production minus the cost of plant respiration, is the source of C in afforested land, and its rate determines the amount of C from aboveground litter fall, root exudates and rhizodeposition that can be potentially sequestered in soils [53]. In comparison with NPP rates reported in other studies [54–56], we found that annual NPP (including trees, shrubs and herbs) was 273.4 g C m^-2^ yr^-1^ (n = 2, SD = 209.1 g C m^-2^ yr^-1^) in shrub afforested sites and 363.5 g C m^-2^ yr^-1^ (n = 11, SD = 192.3 g C m^-2^ yr^-1^) in tree-dominated afforested sites on the Qinghai Plateau (S2 Table). In total, SOC accumulation in shrub- and tree-dominated afforested sites accounted for 34% and 24%, respectively, of the total C sink of forest ecosystems in this region, a lower sequestration amount than the 36% observed in the larch plantations of Northeastern China [49]. Therefore, although the rates of SOC accumulation following afforestation on the Qinghai Plateau were higher than those previously reported at the global and/or regional scale, these rates were still relatively comparable.

### Effects of Afforestation on Soil TN and Its Associations with SOC

Nitrogen dynamics are a key factor in the regulation of C sequestration in terrestrial ecosystems [21,57]. Soil N losses typically occur in the soil of afforested areas, owing to depletion associated with forest growth [45,49]. In Contrast, high levels of N sequestration, even up to 15.3 g N m^-2^ yr^-1^ in soil depths of 0–50 cm depth, were observed in the afforested land containing N-fixer species [19,24]. Afforested soils may gain N mainly from three sources: biological N fixation, atmospheric N deposition and fertilization [18,21]. In this study, the average rate of TN stock change (soil depths of 0–60 cm) was 4.6 g N m^-2^ yr^-1^ for shrub-dominated afforested sites and 6.7 g N m^-2^ yr^-1^ for tree-dominated afforested sites (Table 1), which is consistent with the value of 9.5 g N m^-2^ yr^-1^ (range: 3–14 g N m^-2^ yr^-1^) found in a recent global meta-analysis examining afforestation effects in the top meter of soil, with site ages of ≥50 years [18]. In Qinghai province, rates of atmospheric N deposition averaged 0.67 g N m^-2^ yr^-1^ and ranged from 0.11–1.78 g N m^-2^ yr^-1^ [58]. The mean rate of biological N fixation in arid shrubland is estimated at 5.6 g N m^-2^ yr^-1^, which is higher than in other ecosystem types [59]. According to forest management records for the study area, the sample afforested plots were not fertilized in the growing season. Thus, we speculated that the higher N accumulation ability of soils observed on the Qinghai Plateau might be explained by greater biological N fixation.

Restoration of degraded land typically increases the number of plant species occurring there and the site biomass productivity [33,57]. Most of our study sites planted N-fixing species such as *Medicago sativa L*. and *Hippophae rhamnoides* as accompanying species to the shrubs or trees, in order to enhance the survival of the dominant species (S1 Table). This inclusion of N-fixing species can lead to greater rates of return organic N to soils through biological N fixation, plant litters and root residues. In this study, the average rate of change in TN stocks in shrub-grass ecosystems was 1.98-fold greater than in pure shrub plantations, while the average rate of change in TN stocks under mixed forest with N-fixers was 3.39-fold greater than in forest without N-fixers (Table 1). Accretion of soil TN involves many processes, including soil C decomposition and stabilization in soil biogeochemistry [19,60]. In this study, a significant relationship was found between the rates of change in SOC and TN stocks in both shrub- and tree-dominated afforested sites (Figs. 4 and 5). Previous studies [19,24] have shown that the inclusion of N-fixers can substantially increase available N via biological nitrogen fixation, which in turn facilitates C sequestration and improves soil fertility in forested land. Although SOC and TN stocks increased synchronously after afforestation in our study, the rate of relative change in SOC stocks was significantly greater than the rate of relative change in TN stocks in both the pure shrub plantations and tree-dominated afforested plots (Figs. 4 and 5). Furthermore, the C/N ratio increased significantly in both shrub- and tree-dominated afforested sites (Fig. 6B). The theory of progressive N limitation predicts that soil C sequestration will not be sustainable if soil mineral N content decreases over time [61]. However, owing to the relatively high rate of N sequestration, we speculated that the soil C sequestration is not likely to be limited by a shortage of soil TN in afforested land on the Qinghai Plateau.

### Factors Affecting Post-Afforestation Changes in SOC and TN

Many biotic and abiotic factors can influence post afforestation changes in SOC and TN contents [17,18]. For example, as discussed above, the particular plant species utilized in afforestation in our study area strongly influenced changes in SOC and TN change in afforested land. The presence of N-fixing species can increase SOC sequestration in afforested soils by decreasing the decomposition rate of C pools, or by increasing humus formation [19,24,62]. In our study area, N-fixers such as *Medicago sativa* and C4-herbs such as *Elymus nutans* were the main accompanying species in shrub-dominated afforested sites. Plant species richness and functional group composition have been shown to influence rates of both SOC and TN decomposition and accumulation in restored ecosystems [63,64]. However, owing to the large amount of variations seen among shrubland sites, the rates of absolute change in both SOC and TN stocks did not differ significantly between shrub-grass ecosystems and pure shrub plantations, with the exception of in TN stocks at soil depths of 0–10 cm (*p* < 0.05) (Table 1). For tree-dominated afforested sites, the rate of change in soil SOC and TN was 1.29-fold and 3.39-fold greater, respectively, in sites with versus without N-fixing species (*p* < 0.05) (Table 1). The N-fixers thus critically influenced SOC and TN accretion in tree-dominated afforested sites, as reported in previous studies [19,24]. Statistical analysis indicated that the slopes of the regression of relative change in SOC content vs. relative change in TN content in mixed forests with N-fixers was significantly greater than that in forests without N-fixers (*p* < 0.01) (Fig. 5B), indicating that greater SOC accumulation capacity existed in forests with versus without N-fixing species.

The afforested sites were relatively low diversity ecosystems, with all plant species, including shrubs and grasses, on afforested land originating either from artificial introductions or natural succession. The plant species richness, which was closely related to mean annual precipitation (*r* = 0.55, *p* < 0.01), was positively related to relative changes in SOC and TN (in the top 20 cm of soil) in tree-dominated afforested sites (Table 2). The impact of plant species richness on SOC and TN accretion rates may be attributed to increases in the productivity of afforested ecosystems and to the interactive effects of N-fixing and dominant afforestation species on soil biochemistry [63–65].

Two additional factors may also contribute the high SOC accumulation rates in our study area. Firstly, higher SOC accumulation ability may be fostered by low initial SOC content in degraded land that is subsequently afforested [66]. In this study, the mean SOC content (top 20 cm of depth) of all reference plots was 24.7 Mg C ha^-1^, which is much lower than the mean level reported globally for cropland (45.9 Mg C ha^-1^) [1]. Forty-two afforested sites were previously used as cropland and had a long-term history of cultivation prior to afforestation (S1 Table), implying the widespread existence of poor soil fertility in the examined afforested land. The increasing input of organic carbon from plant litter and root residues after restoration will be retained as particulate organic matter (POM) or fixed by mineral particles, contributing to SOC sequestration [60,67]. Additionally, land restoration reduces erosions by water and wind, both of which can cause a severe depletion of the SOC pool [5]. Secondly, the SOC sequestration after afforestation has been shown to be affected by climatic zone [17,18]. With its unique plateau climate, the mean annual temperature for Qinghai Province is 8–12°C lower than the average for the same latitude on the North China Plain [30]. The decomposition rate of SOC from plant litters and root residues is lower in cooler environments compared to temperate and tropical zones [68]. Additionally, nearly 75% of the yearly precipitation for the Qinghai Plateau occurs in the summer, owing to the Indian ocean-monsoon [69], which can be beneficial to plant growth; furthermore, cold temperatures outside of the growing season can decrease the decomposition rate of the C pool.

### Implications for SOC Sequestration and Uncertainties

China’s program of returning farmland to forest is one of the largest such attempts in the world. Compared to C sequestration in biomass, the amount of C sequestration in soils is very difficult to measure [50]. Additionally, knowledge of whether afforestation will cause a reduction in soil fertility is critical for the sustainable development of forests [49]. This systematical investigation revealed that there was a high capacity for SOC and TN sequestration (in both top- and deep-soil layers) after afforestation. The rate of SOC sequestration in the top 60 cm of soil in Qinghai province was estimated at 0.27 Tg C yr^-1^ for shrub-dominated afforested sites and 0.29 Tg C yr^-1^ for tree-dominated afforestation systems (based on an approximation where SOC sequestration = area × rate of SOC accumulation, where the area afforested with shrubs or trees was the relevant proportion of the total afforestation area). Piao et al. [70], using process-based models, estimated that the soil C sink corresponds to 43% of the total terrestrial C sink of China. Our results supply essential information for evaluating of the carbon sequestration potential of forestry projects implemented over the past few decades. At a regional scale, this study revealed that the mineral soils acted as a C sink following afforestation in Qinghai-Tibet Plateau zone, a region about which little is known on the effects of afforestation. Afforestation also had a positive effect on TN storage at soil depths of 0–60 cm and soil BD decreased with afforestation age (Table 1 and Fig. 6A), suggesting the soil fertility was improved by afforestation and that SOC is unlikely to be limited by low N availability in afforested soils. Therefore, this study on the Qinghai Plateau provides crucial information for understanding the mechanisms underlying changes in SOC and TN following afforestation at a regional scale.

Although we collected 15 soil cores at each afforested site, in order to reduce the bias introduced by spatial heterogeneity (Fig. 2), there remains some limitations in the interpretation of the results of this study. Firstly, the paired-plots method we used is based on the ecological theory of “space for time”, which assumed that the initial SOC content before restoration was similar across sites under the same abiotic conditions. The disadvantages of the paired-plots method are obvious: when there is high spatial variability in SOC and no data available on how SOC changes (over time) in reference sites, a lack of initial SOC content baseline measurements for specific sites will cause errors [71]. It has been estimated that the paired-plots method overestimates changes in SOC stocks by 12.4% compared to the retrospective method [17]. Secondly, as was reflected in our multiple regression models (Table 2), the response variables in shrub-dominated afforested sites were poorly fitted in comparison to the tree-dominated afforested sites. The poor model fit may be caused by the great spatial variations in SOC content, TN content and BD reported at fine scales in shrub-dominated afforestation systems [72,73]. Unlike in the tree plantations examined here, the shrub species used in afforestation, e.g. *Hippophae rhamnoides* and *Caragana intermedia*, clustered spatially in distinctive groups in the field, with lower and smaller canopies (than the tree canopies) (Fig. 1B). In a semi-desert grassland, SOC and TN contents were not affected beyond the shrub canopy of *Prosopis velutina* encroachment [73]. Lastly, the use of completely random sampling may not produce reliable estimates of SOC sequestration in shrub encroachments in grassland, as this sampling design ignores the strong spatial patterns in SOC and TN contents, derived from differences in shrub size and subcanopy location [72–74]. If such strong spatial patterning also occurs in shrub plantations, then current sampling strategies need to be redesigned in order to get more accurate estimates of SOC sequestration.

## Conclusions

The results of our systematic investigation showed that SOC increase dramatically after afforestation on the Qinghai Plateau. The rate of change in SOC was strongly correlated with the rate of change in TN in tree-dominated afforestation plots. Soil TN increased synchronously with SOC sequestration on the Qinghai Plateau. Afforestation age and number of total plant species combined explained more than 75% of the variance in SOC content change after planting trees on the Qinghai Plateau. However, these explanatory variables did not fully characterize changes in SOC content observed after planting shrubs. As soil TN increased with afforestation ages and the soil BD decreased with afforestation ages, we concluded that afforestation might improve soil fertility to some extent in our system. Finally, this study provides much needed information on the pattern of SOC accumulation following afforestation in a plateau climate, and provides new evidence regarding the C sequestration potential associated with forestry projects in China.

## Supporting Information

S1 Fig The accretion rates of SOC and TN stocks in forest floor at four typical afforestation sites on the Qinghai Plateau. (DOCX)

S1 Table Basic information on soil sample sites. (DOCX)

S2 Table Net primary productivity under different afforestation types on the Qinghai Plateau. (DOCX)

## References

[1] Jobbágy EG, Jackson RB (2000) The vertical distribution of soil organic carbon and its relation to climate and vegetation. Ecological Applications 10: 423–436.

[2] Guo L, Gifford R (2002) Soil carbon stocks and land use change: a meta analysis. Global Change Biology 8: 345–360.

[3] IPCC (2007) The Physical Science Basis Contribution of Working Group I to the Fourth Assessment Report of the Intergovernmental Panel on Climate Change. In: Cambridge University Press, Cambridge, United Kingdom and New York, NY, USA.

[4] Houghton RA (2007) Balancing the global carbon budget. Annual Review of Earth and Planetary Sciences 35: 313–347

[5] Lal R (2003) Soil erosion and the global carbon budget. Environment International 29: 437–450. 12705941 10.1016/S0160-4120(02)00192-7

[6] Pan Y, Birdsey RA, Fang J, Houghton R, Kauppi PE, et al (2011) A large and persistent carbon sink in the world’s forests. Science 333: 988–993. 10.1126/science.1201609 21764754

[7] Chang R, Fu B, Liu G, Liu S (2011) Soil Carbon Sequestration Potential for “Grain for Green” Project in Loess Plateau, China. Environmental Management 48: 1158–1172. 10.1007/s00267-011-9682-8 21553107

[8] Jarecki MK, Lal R (2003) Crop management for soil carbon sequestration. Critical Reviews in Plant Sciences 22: 471–502.

[9] Six J, Frey SD, Thiet RK, Batten KM (2006) Bacterial and fungal contributions to carbon sequestration in agroecosystems. Soil Science Society of America Journal 70: 555–569.

[10] Tang JW, Bolstad PV, Martin JG (2009) Soil carbon fluxes and stocks in a Great Lakes forest chronosequence. Global Change Biology 15: 145–155.

[11] Bárcena TG, Kiær LP, Vesterdal L, Stefánsdóttir HM, Gundersen P, et al (2014) Soil carbon stock change following afforestation in Northern Europe: a meta‐analysis. Global Change Biology 20: 2393–2405. 10.1111/gcb.12576 24634314

[12] Song XZ, Peng CH, Zhou GM, Jiang H, Wang WF (2014) Chinese Grain for Green Program led to highly increased soil organic carbon levels: A meta-analysis. Scientific Reports 4. 10.1038/srep04460 PMC3967516 24675818

[13] Paul K, Polglase P, Nyakuengama J, Khanna P (2002) Change in soil carbon following afforestation. Forest Ecology and Management 168: 241–257.

[14] Zhang K, Dang H, Tan S, Cheng X, Zhang Q (2010) Change in soil organic carbon following the ‘Grain‐for‐Green’programme in China. Land Degradation & Development 21: 13–23.

[15] Shi SW, Zhang W, Zhang P, Yu YQ, Ding F (2013) A synthesis of change in deep soil organic carbon stores with afforestation of agricultural soils. Forest Ecology and Management 296: 53–63.

[16] Don A, Schumacher J, Freibauer A (2011) Impact of tropical land-use change on soil organic carbon stocks—a meta-analysis. Global Change Biology 17: 1658–1670.

[17] Laganière J, Angers Da, Paré D (2010) Carbon accumulation in agricultural soils after afforestation: a meta‐analysis. Global Change Biology 16: 439–453.

[18] Li DJ, Niu SL, Luo YQ (2012) Global patterns of the dynamics of soil carbon and nitrogen stocks following afforestation: a meta-analysis. New Phytologist 195: 172–181. 10.1111/j.1469-8137.2012.04150.x 22512731

[19] Binkley D (2005) How nitrogen-fixing trees change soil carbon In: Binkley D, Menyailo O, editors. Tree Species Effects on Soils: Implications For Global Change. Dordrecht: Springer pp. 155–164.

[20] Smith P, Davies CA, Ogle S, Zanchi G, Bellarby J, et al (2012) Towards an integrated global framework to assess the impacts of land use and management change on soil carbon: current capability and future vision. Global Change Biology 18: 2089–2101.

[21] Liu L, Greaver TL (2010) A global perspective on belowground carbon dynamics under nitrogen enrichment. Ecology Letters 13: 819–828. 10.1111/j.1461-0248.2010.01482.x 20482580

[22] Luo YQ, Field CB, Jackson RB (2006) Does nitrogen constrain carbon cycling, or does carbon input stimulate nitrogen cycling? Ecology 87: 3–4.

[23] Neff JC, Townsend AR, Gleixner G, Lehman SJ, Turnbull J, et al (2002) Variable effects of nitrogen additions on the stability and turnover of soil carbon. Nature 419: 915–917. 12410307 10.1038/nature01136

[24] Resh SC, Binkley D, Parrotta JA (2002) Greater Soil Carbon Sequestration under Nitrogen-fixing Trees Compared with Species. Ecosystems 5: 0217–0231.

[25] Bowden RD, Davidson E, Savage K, Arabia C, Steudler P (2004) Chronic nitrogen additions reduce total soil respiration and microbial respiration in temperate forest soils at the Harvard Forest. Forest Ecology and Management 196: 43–56.

[26] Wang SL, Zhang WD, Sanchez F (2010) Relating net primary productivity to soil organic matter decomposition rates in pure and mixed Chinese fir plantations. Plant and Soil 334: 501–510.

[27] Yang Y, Luo Y, Finzi AC (2011) Carbon and nitrogen dynamics during forest stand development: a global synthesis. New Phytologist 190: 977–989. 10.1111/j.1469-8137.2011.03645.x 21323927

[28] Morris SJ, Bohm S, Haile-Mariam S, Paul EA (2007) Evaluation of carbon accrual in afforested agricultural soils. Global Change Biology 13: 1145–1156.

[29] Hoogmoed M, Cunningham SC, Thomson JR, Baker PJ, Beringer J, et al (2012) Does afforestation of pastures increase sequestration of soil carbon in Mediterranean climates? Agriculture Ecosystems & Environment 159: 176–183.

[30] Lin ZY, Wu XD (1981) Cumatic regionalization of the Qinghai-Xizang Plateau. Acta Geographica Sinica 1: 22–32.

[31] Poeplau C, Don A, Vesterdal L, Leifeld J, Van Wesemael BAS, et al (2011) Temporal dynamics of soil organic carbon after land-use change in the temperate zone—carbon response functions as a model approach. Global Change Biology 17: 2415–2427.

[32] Powers JS, Corre MD, Twine TE, Veldkamp E (2011) Geographic bias of field observations of soil carbon stocks with tropical land-use changes precludes spatial extrapolation. Proceedings of the National Academy of Sciences of the United States of America 108: 6318–6322. 10.1073/pnas.1016774108 21444813 PMC3076837

[33] Dong X (2011) Effects of grain-for-green on soil quality in Huangshui River Basin of Qinghai Province. Bulletin of Soil and Water Conservation 31: 45–48.

[34] Fan GH, Wang HL (2008) The effect of restoration on soil organic matters, pH and minreal materials in Ping-an, Qinghai province. Qinghai Science and Technology 1: 17–19.

[35] State Forestry Administration PCC (2010) China Forestry Statistical yearbook. Beijing: China Forestry Publishing House pp. 498.

[36] Ellert B, Bettany J (1995) Calculation of organic matter and nutrients stored in soils under contrasting management regimes. Canadian Journal of Soil Science 75: 529–538.

[37] Bowman R, Reeder J, Wienhold B (2002) Quantifying laboratory and field variability to assess potential for carbon sequestration. Communications in soil science and plant analysis 33: 1629–1642.

[38] Yang Y, Mohammat A, Feng J, Zhou R, Fang J (2007) Storage, patterns and environmental controls of soil organic carbon in China. Biogeochemistry 84: 131–141.

[39] Hedges LV, Gurevitch J, Curtis PS (1999) The meta-analysis of response ratios in experimental ecology. Ecology 80: 1150–1156.

[40] Huxley R, Neil A, Collins R (2002) Unravelling the fetal origins hypothesis: is there really an inverse association between birthweight and subsequent blood pressure? Lancet 360: 659–665. 12241871 10.1016/S0140-6736(02)09834-3

[41] Zillich AJ, Garg J, Basu S, Bakris GL, Carter BL (2006) Thiazide diuretics, potassium, and the development of diabetes—A quantitative review. Hypertension 48: 219–224. 16801488 10.1161/01.HYP.0000231552.10054.aa

[42] Saiz G, Bird MI, Domingues T, Schrodt F, Schwarz M, et al (2012) Variation in soil carbon stocks and their determinants across a precipitation gradient in West Africa. Global Change Biology 18: 1670–1683.

[43] Berry SL, Roderick ML (2002) Estimating mixtures of leaf functional types using continental-scale satellite and climatic data. Global Ecology and Biogeography 11: 23–39.

[44] Eclesia RP, Jobbagy EG, Jackson RB, Biganzoli F, Pineiro G (2012) Shifts in soil organic carbon for plantation and pasture establishment in native forests and grasslands of South America. Global Change Biology 18: 3237–3251. 28741815 10.1111/j.1365-2486.2012.02761.x

[45] Berthrong ST, Jobbágy EG, Jackson RB (2009) A global meta-analysis of soil exchangeable cations, pH, carbon, and nitrogen with afforestation. Ecological Applications 19: 2228–2241. 20014590 10.1890/08-1730.1

[46] Mao R, Zeng DH (2010) Changes in Soil Particulate Organic Matter, Microbial Biomass, and Activity Following Afforestation of Marginal Agricultural Lands in a Semi-Arid Area of Northeast China. Environmental Management 46: 110–116. 10.1007/s00267-010-9504-4 20508936

[47] Richter DD, Markewitz D, Trumbore SE, Wells CG (1999) Rapid accumulation and turnover of soil carbon in a re-establishing forest. Nature 400: 56–58.

[48] Hansen EA (1993) SOIL carbon sequestration beneath hybrid poplar plantations in the north central united-states. Biomass & Bioenergy 5: 431–436.

[49] Wang WJ, Qiu L, Zu YG, Su DX, An J, et al (2011) Changes in soil organic carbon, nitrogen, pH and bulk density with the development of larch (Larix gmelinii) plantations in China. Global Change Biology 17: 2657–2676.

[50] McKinley D, Ryan M, Birdsey R, Giardina C, Harmon M, et al (2011) A synthesis of current knowledge on forests and carbon storage in the United States. Ecological Applications 21: 1902–1924. 21939033 10.1890/10-0697.1

[51] Janssens IA, Freibauer A, Ciais P, Smith P, Nabuurs GJ, et al (2003) Europe’s terrestrial biosphere absorbs 7 to 12% of European anthropogenic CO2 emissions. Science 300: 1538–1542. 12764201 10.1126/science.1083592

[52] Zhou XH, Zhou T, Luo YQ (2012) Uncertainties in carbon residence time and NPP-driven carbon uptake in terrestrial ecosystems of the conterminous USA: a Bayesian approach. Tellus Series B-Chemical and Physical Meteorology 64.

[53] Pineiro G, Oesterheld M, Batista WB, Paruelo JM (2006) Opposite changes of whole-soil vs. pools C: N ratios: a case of Simpson’s paradox with implications on nitrogen cycling. Global Change Biology 12: 804–809.

[54] Gao G, Li D, Jia J, Hu W, Liu G (2007) Research on soil fertility of different species arrangement models in converted farmland land. Journal of Arid Land Resources and Environment 21, 104–1075.

[55] Lu F (2007) The characteristics of habitat succession and its evaluation of different vegetations in datong county. Ph.D Thesis, Beijing Forestry University. Beijing.

[56] Jing W, Liu X, Zhao W, Ma J (2011) Study On biomass and net prod uctivity of typical forest stand in the Qilian Mountains. Journal of Gansu Gricultural University 6, 81–8.

[57] Knops JMH, Bradley KL (2009) Soil Carbon and Nitrogen Accumulation and Vertical Distribution across a 74-Year Chronosequence. Soil Science Society of America Journal 73: 2096–2104.

[58] Lu CQ, Tian HQ (2007) Spatial and temporal patterns of nitrogen deposition in China: Synthesis of observational data. Journal of Geophysical Research-Atmospheres 112: D22S05.

[59] Cleveland CC, Townsend AR, Schimel DS, Fisher H, Howarth RW, et al (1999) Global patterns of terrestrial biological nitrogen (N-2) fixation in natural ecosystems. Global Biogeochemical Cycles 13: 623–645.

[60] Giardina CP, Binkley D, Ryan MG, Fownes JH, Senock RS (2004) Belowground carbon cycling in a humid tropical forest decreases with fertilization. Oecologia 139: 545–550. 15071736 10.1007/s00442-004-1552-0

[61] Luo Y, Su B, Currie WS, Dukes JS, Finzi AC, et al (2004) Progressive nitrogen limitation of ecosystem responses to rising atmospheric carbon dioxide. Bioscience 54: 731–739.

[62] Kaye JP, Resh SC, Kaye MW, Chimner RA (2000) Nutrient and carbon dynamics in a replacement series of Eucalyptus and Albizia trees. Ecology 81: 3267–3273.

[63] Fornara DA, Tilman D (2008) Plant functional composition influences rates of soil carbon and nitrogen accumulation. Journal of Ecology 96: 314–322.

[64] Tilman D, Hill J, Lehman C (2006) Carbon-negative biofuels from low-input high-diversity grassland biomass. Science 314: 1598–1600. 17158327 10.1126/science.1133306

[65] Tilman D, Lehman CL, Thomson KT (1997) Plant diversity and ecosystem productivity: theoretical considerations. Proceedings of the National Academy of Sciences 94: 1857–1861. 11038606 10.1073/pnas.94.5.1857 PMC20007

[66] Lugo AE, Brown S (1993) Management of tropical soils as sinks or sources of atmospheric carbon. Plant and Soil 149: 27–41.

[67] Del Galdo I, Six J, Peressotti A, Francesca Cotrufo M (2003) Assessing the impact of land‐use change on soil C sequestration in agricultural soils by means of organic matter fractionation and stable C isotopes. Global Change Biology 9: 1204–1213.

[68] Chapin FS III, Matson PA, Vitousek PM (2011) Principles of terrestrial ecosystem ecology: Springer.

[69] Huang Y, Wang B, Wang P (2006) Analysis of summer precipitation anomaly and water vapor transport in Qinghai Plateau (in chinese). Meteorological: 18–23.

[70] Piao S, Fang J, Ciais P, Peylin P, Huang Y, et al (2009) The carbon balance of terrestrial ecosystems in China. Nature 458: 1009–1013. 10.1038/nature07944 19396142

[71] Hoover CM (2003) Soil carbon sequestration and forest management: challenges and opportunities The Potential of US Forest Soils to Sequester Carbon and Mitigate the Greenhouse Effect CRC Press, Boca Raton, FL: 211–238.

[72] Liu F, Wu XB, Bai E, Boutton TW, Archer SR (2010) Spatial scaling of ecosystem C and N in a subtropical savanna landscape. Global Change Biology 16: 2213–2223.

[73] Throop HL, Archer SR (2008) Shrub (Prosopis velutina) encroachment in a semidesert grassland: spatial-temporal changes in soil organic carbon and nitrogen pools. Global Change Biology 14: 2420–2431.

[74] Liu F, Wu XB, Bai E, Boutton TW, Archer SR (2011) Quantifying soil organic carbon in complex landscapes: an example of grassland undergoing encroachment of woody plants. Global Change Biology 17: 1119–1129.

