# Supplementary material for: The Impact of Afforestation on Soil Organic Carbon Sequestration on the Qinghai Plateau, China
Source: PLoS One. 2015 Feb 23;10(2):e0116591. doi: 10.1371/journal.pone.0116591 (PMC4338072; doi:10.1371/journal.pone.0116591)
Supplement: S1 Fig — (DOCX) [file pone.0116591.s001.docx]

Figure S1. The accretion rate of C and TN stocks in forest floor at 4 typical afforestation sites on the Qinghai Plateau.

The number of 1~4 at X-axis represents a 57-year *Populus cathayana Lauche* forest, a 33-year *Populus cathayana Rehd.* forest, a 26-year *Larix principis-rupprechtii* forest, and a 29-year *Larix principis-rupprechtii* forest, respectively. It should be informed that accretion rate of C and TN stocks in forest floor from these sites (e.g sites of 1 and 2) was much less accurate than that in mineral soil layers due to lack of [reduplicate](app:ds:reduplicate)s in sample sizes.
