# Supplementary material for: The Impact of Afforestation on Soil Organic Carbon Sequestration on the Qinghai Plateau, China
Source: PLoS One. 2015 Feb 23;10(2):e0116591. doi: 10.1371/journal.pone.0116591 (PMC4338072; doi:10.1371/journal.pone.0116591)
Supplement: S1 Table — (DOCX) [file pone.0116591.s002.docx]

Table S1.

Basic information on soil sample sites.

| Site | Soil Type | | Previous land use | Water Status | Ele* | | MAP^+^ | | MAT^Δ^ | | Soil Texture  (0-20cm) | Forest type | Dominated species | Accompanying species | Forest floor depths (cm)^§^ | Age^#^ | Georeference position |
| --- | --- | --- | --- | --- | --- | --- | --- | --- | --- | --- | --- | --- | --- | --- | --- | --- | --- |
| DT1 | | Castanozem | Cropland(highland barley) | Rainfed | | 2969 | | 556 | 2.9 | Clay 9% and sand 18.7% | | shrubs | *Hippophae rhamnoides* | *Elymus dahuricus* | no | 6 | 101°51.82'E, 37°04.93N |
| DT2 | | Castanozem | Cropland(highland barley) | Rainfed | | 2971 | | 556 | 2.9 | Clay 6.5% and sand 26.3% | | shrubs | *Hippophae rhamnoides* | *Elymus dahuricus* | no | 9 | 101°51.70'E 37°04.97N |
| MY | | Castanozem | Cropland(potato) | Rainfed | | 1733 | | 523 | 0.9 | Clay 6.3% and sand 33.8% | | shrubs | *Hippophae rhamnoides* | | no | 8 | 101°46.78′E 37°20.67N |
| HZ1 | | Castanozem | Cropland(wheat-rape) | Rainfed | | 2826 | | 650 | 0.9 | Clay 7% and sand 24% | | shrubs | *Hippophae rhamnoides* | | no | 11 | 101°53.98′E 36°58.72N |
| HZ3 | | Castanozem | Cropland(wheat-rape) | Rainfed | | 2867 | | 650 | 0.8 | Clay 3% and sand 41.4% | | tree | *Larix principis-rupprechtii* | *Salix oritrepha,*  *Thalictrum aquilegifolium,*  and *Fragaria orientalis* | 3~4 cm | 29 | 101°53.60′E 36°58.52N |
| HZ4 | | Castanozem | Cropland(wheat-rape) | Rainfed | | 2796 | | 650 | 0.9 | Clay 5.4% and sand 34.9% | | shrubs | *Hippophae rhamnoides* | *Picea crassifolia, Elymus dahuricus* | no | 3 | 101°53.60′E 36°58.57N |
| HZ5 | | Castanozem | Cropland(wheat) | Rainfed | | 2806 | | 350 | 0.9 | Clay 4.7% and sand40.3% | | shrubs | *Caragana intermedia* | *Medicago sativa L.* | no | 9 | 101°51.43′E 36°44.07N |
| PA3 | | Gley | Cropland(wheat) | Rainfed | | 2983 | | 450 | 1.4 | Clay 7.1% and sand 25.7% | | tree | *Larix principis-rupprechtii* | | 2~3 cm | 26 | 102°04.10′E 36°18.22N |
| HY1 | | Castanozem | Cropland(rape) | Rainfed | | 3068 | | 400 | 3 | Clay 6.3% and sand 28.3% | | shrubs | *Hippophae rhamnoides* | *Carex tristachya* | 2 cm | 23 | 101°12.33′E 36°35.53N |
| HY3 | | Castanozem | Cropland(rape) | Rainfed | | 3064 | | 400 | 3 | Clay 6.1% and sand 31.8% | | tree | *Betula platyphylla* | *Carex tristachya* | 1~2 cm | 10 | 101°12.32′E 36°35.58N |
| HaiY1 | | Castanozem | Cropland(rape) | Rainfed | | 3001 | | 350 | 0.2 | Clay 4.5% and sand 51.3% | | shrubs | *Hippophae rhamnoides* | *Stipa purpurea* | no | 14 | 101°12.35′E 36°35.63N |
| HaiY2 | | Castanozem | Cropland(rape) | Rainfed | | 3001 | | 350 | 0.2 | Clay 6.4% and sand 34.7% | | shrubs | *Hippophae rhamnoides* | | no | 29 | 101°01.05′E 36°52.42N |
| HaiY3 | | Castanozem | Cropland(rape) | Rainfed | | 3001 | | 350 | 0.2 | Clay 4.3% and sand 39.2% | | shrubs | *Salix sclerophylla* | | no | 29 | 101°01.05′E 36°52.50N |
| GC1 | | Meadow soil | Cropland(rape) | Irrigated | | 3254 | | 380 | -1.3 | Clay 5.2% and sand 38.5% | | shrubs | *Hippophae rhamnoides* | *Elymus dahuricus* | no | 9 | 99°53.25′E 37°16.13N |
| GC2 | | Meadow soil | Cropland(rape) | Irrigated | | 3284 | | 327 | -1.2 | Clay 4.7% and sand 42.3% | | shrubs | *Hippophae rhamnoides* | *Elymus dahuricus* | no | 9 | 101°26.07′E 37°14.55N |
| GC3 | | Meadow soil | Cropland(rape) | Irrigated | | 3231 | | 327 | -1.2 | Clay 7.7% and sand 24.2% | | shrubs | *Hippophae rhamnoides* | *Medicago sativa L.* | no | 8 | 101°24.27′E 37°12.63N |
| LD1 | | Castanozem | Cropland(potato) | Rainfed | | 2528 | | 330 | 7.3 | Clay 5.9% and sand 37.2% | | tree | *Hippophae rhamnoides* | | 1~2 cm | 11 | 102°21.92′E 36°33.82N |
| LD2 | | Castanozem | Cropland(potato) | Rainfed | | 2545 | | 330 | 7.3 | Clay 5.9% and sand 34.4% | | tree | *Ulmus pumila* | | no | 8 | 102°21.82′E 36°33.87N |
| LD3 | | Castanozem | Cropland(potato) | Rainfed | | 2521 | | 330 | 7.3 | Clay 5.4% and sand 41.1% | | shrubs | *Ulmus pumila* | | no | 11 | 102°21.88′E 36°33.92N |
| LD4 | | Castanozem | Cropland(potato) | Rainfed | | 2456 | | 330 | 7.3 | Clay 5.7% and sand 38.1% | | tree | *Populus bolleana* |  | no | 11 | 102°21.97′E 36°34.17N |
| DGL1 | | Grey-brown desert soil | Cropland(wheat) | Irrigated | | 2769 | | 38 | 3.6 | Clay 5% and sand 51.1% | | tree | *Populus bolleana* | *Medicago sativa L.* | no | 9 | 95°42.70′E 36°26.45N |
| DGL2 | | Grey-brown desert soil | Cropland(wheat) | Irrigated | | 2775 | | 38 | 3.6 | Clay 5.6% and sand 45.7% | | tree | *Populus bolleana* | *Medicago sativa L.* | no | 9 | 95°44.73′E  36°26.62N |
| GEM1 | | Grey-brown desert soil | Cropland(wheat) | Irrigated | | 2817 | | 41 | 4.3 | Clay 6.7% and sand 38.6% | | shrubs | *Hippophae rhamnoides* | | no | 9 | 94°48.52′E 36°25.77N |
| GEM2 | | Grey-brown desert soil | Cropland(wheat) | Irrigated | | 2817 | | 41 | 4.3 | Clay 3.7% and sand 48.6% | | shrubs | *Salix sclerophylla* | | no | 9 | 94°48.62′E 36°25.75N |
| NC1 | | Grey-brown desert soil | Cropland(wheat) | Irrigated | | 2821 | | 41 | 4.3 | Clay 5% and sand 50.7% | | shrubs | *Lycium barbarum* | | no | 8 | 95°01.50′E 36°42.35N |
| NC2 | | Grey-brown desert soil | Cropland(wheat) | Irrigated | | 2821 | | 41 | 4.3 | Clay 2.1% and sand 78.3% | | shrubs | *Elaeagnus angustifolia* | | no | 8 | 95°01.55′E 36°42.40N |
| DLH1 | | Sandy soil | Cropland(wheat) | Irrigated | | 2874 | | 185 | 2.7 | Clay 2.6% and sand 75.7% | | tree | *Populus bolleana* |  | no | 3 | 97°27.07′E 37°13.68N |
| DLH2 | | Grey-brown desert soil | Cropland(wheat) | Irrigated | | 2882 | | 90 | 3.5 | Clay 5.1% and sand 40.1% | | tree | *Elaeagnus angustifolia* | *Medicago sativa L.* | no | 9 | 96°44.68′E 37°20.70N |
| WL1 | | Brown soil | Cropland(wheat) | Irrigated | | 2953 | | 180 | 4.2 | Clay 5.6% and sand 42.5% | | shrubs | *Hippophae rhamnoides* | *Medicago sativa L.* | no | 9 | 98°34.17′E 37°01.37N |
| WL2 | | Brown soil | Cropland(wheat) | Irrigated | | 2978 | | 180 | 4.2 | Clay 5.1% and sand 48.6% | | tree | *Populus bolleana* | *Hippophae rhamnoides,* | no | 10 | 98°21.80′E 36°55.85N |
| WL3 | | Brown soil | Cropland(rape) | Irrigated | | 3225 | | 180 | 4.2 | Clay 5.7% and sand 36.6% | | shrubs | *Hippophae rhamnoides* | *Medicago sativa L.* | no | 9 | 98°34.17′E 37°01.37N |
| WL4 | | Brown soil | Cropland(rape) | Irrigated | | 3272 | | 180 | 4.2 | Clay 5.5% and sand 36.8% | | tree | *Populus bolleana* |  | 1~2 cm | 21 | 98°34.08′E 37°01.90N |
| DL1 | | Brown soil | Cropland(highland barley) | Irrigated | | 3223 | | 198 | 2.7 | Clay 5.6% and sand 38.3% | | tree | *Populus bolleana* |  | 2~3 cm | 27 | 98°06.23′E 36°15.70N |
| DL2 | | Brown soil | Cropland(highland barley) | Irrigated | | 3223 | | 198 | 2.7 | Clay 4.5% and sand 40.5% | | tree | *Populus cathayana Rehd.* |  | 2~3 cm | 33 | 98°06.20′E 37°15.67N |
| DL3 | | Brown soil | Cropland(highland barley) | Irrigated | | 3201 | | 198 | 2.7 | Clay 4.4% and sand 44.5% | | tree | *Populus bolleana* | *Medicago sativa L.* | no | 9 | 98°34.08′E 37°01.90N |
| XRD | | Brown soil | Cropland(wheat) | Irrigated | | 3068 | | 168 | 3.9 | Clay 3.9% and sand 43.1% | | tree | *Populus bolleana* |  | no | 8 | 97°50.08′E 35°59.88N |
| RMH1 | | Sandy soil | Barren land | Irrigated | | 2789 | | 43 | 4.3 | Clay 4.9% and sand 45.9% | | tree | *Populus alba var. pyramidalis* |  | no | 4 | 96°27.27′E 36°21.58N |
| RMH2 | | Sandy soil | Barren land | Irrigated | | 2789 | | 43 | 4.3 | Clay 3.5% and sand 63% | | tree | *Populus alba var. pyramidalis* | *Agropyron cristatum* | 1~2 cm | 21 | 96°27.97′E 36°24.70N |
| RMH4 | | Sandy soil | Barren land | Irrigated | | 2784 | | 43 | 4.3 | Clay 3.5% and sand 63% | | tree | *Populus alba var. pyramidalis* |  | 2~3 cm | 31 | 96.13°27′E 36°24.67N |
| RMH5 | | Sandy soil | Barren land | Irrigated | | 2781 | | 43 | 4.3 | Clay 5.5% and sand 55% | | tree | *Populus cathayana Lauche* | *Agropyron cristatum* | 2~3 cm | 57 | 96.23°27′E 36°24.57N |
| GH2 | | Sandy soil | Barren land | Irrigated | | 2922 | | 318 | 4 | Clay 0.3% and sand 93.8% | | shrubs | *Caragana intermedia* | *Agropyron cristatum* | no | 11 | 100°32.05′E 36°12.38N |
| GN1 | | Castanozem | Cropland(highland barley) | Irrigated | | 3129 | | 430 | 2.9 | Clay 4.8% and sand 41.7% | | shrubs | *Hippophae rhamnoides* | *Agropyron cristatum* | no | 9 | 100°44.22′E 35°35.17N |
| GN3 | | Castanozem | Cropland(highland barley) | Irrigated | | 3130 | | 430 | 2.9 | Clay 3.6% and sand 47.5% | | tree | *Populus bolleana* |  | no | 10 | 100°44.28′E 35°35.12N |
| GN4 | | Castanozem | Cropland(highland barley) | Irrigated | | 3130 | | 430 | 2.9 | Clay 4.1% and sand 52.6% | | tree | *Populus bolleana* |  | 2~3 cm | 31 | 100°44.33′E 35°35.10N |
| GD1 | | Castanozem | Cropland(wheat) | Irrigated | | 2285 | | 252 | 7.2 | Clay 5.4% and sand 41.9% | | shrubs | *Armeniaca sibirica* | | no | 13 | 101°28.72′E 36°02.62N |
| GD2 | | Castanozem | Cropland(wheat) | Irrigated | | 2285 | | 252 | 7.2 | Clay 5.7% and sand 41.6% | | tree | *Populus cathayana* | *Hippophae rhamnoides* | no | 13 | 101°28.72′E 36°02.58N |
| GD3 | | Castanozem | Cropland(wheat) | Irrigated | | 2285 | | 252 | 7.2 | Clay 7% and sand 37.7% | | tree | *Picea crassifolia* | *Caragana intermedia* | 1 cm | 13 | 101°28.72′E 36°02.65N |
| GD4 | | Castanozem | Cropland(wheat) | Irrigated | | 2285 | | 252 | 7.2 | Clay 5.1% and sand 45.1% | | shrubs | *Syringa oblata* | *Elaeagnus angustifolia* | no | 13 | 101°28.65′E 36°02.57N |

* Elevation (m)

+ is mean annual precipitation (mm),

Δ is mean annual temperature (℃),

§is depths of forest floor, *no* means no clear evidence of the presence of forest floor ( < 1cm) in forested plots based on direct observation in the field,

# is afforestation age (year).
