# Supplementary material for: The Impact of Afforestation on Soil Organic Carbon Sequestration on the Qinghai Plateau, China
Source: PLoS One. 2015 Feb 23;10(2):e0116591. doi: 10.1371/journal.pone.0116591 (PMC4338072; doi:10.1371/journal.pone.0116591)
Supplement: S2 Table — (DOCX) [file pone.0116591.s003.docx]

Table S2.

The Net Primary Productivity (NPP) under different afforestation modes on Qinghai Plateau

| No. | Location | Tem^Δ^ | pre^+^ | species | Age | NPP*  (Mg C ha^-1^ yr^-1^) | Source |
| --- | --- | --- | --- | --- | --- | --- | --- |
| 1 | Beichaunghe, Datong | 2.8 | 508 | *Populus bolleana+* Hippophae rhamnoides | 5 | 1.99 | (Gao *et al.*, 2010) |
| 2 | Beichaunghe, Datong | 2.8 | 508 | Hippophae rhamnoides | 5 | 4.21 | (Gao *et al.*, 2010) |
| 3 | Beichaunghe, Datong | 2.8 | 508 | Caragana intermedia | 5 | 1.26 | (Gao *et al.*, 2010) |
| 4 | Beichaunghe, Datong | 2.8 | 508 | *Picea crassifolia* | 20 | 6.89 | (Gao *et al.*, 2010) |
| 5 | Beichaunghe, Datong | 2.8 | 508 | Larix principis-ru-pprechtii | 20 | 6.34 | (Gao *et al.*, 2010) |
| 6 | Beichaunghe, Datong | 2.8 | 508 | *Betula platyphylla+ Picea crassifolia* | 20 | 3.10 | (Gao *et al.*, 2010) |
| 7 | Beichaunghe, Datong | 2.8 | 508 | *Picea crassifolia+* Hippophae rhamnoides | 20 | 3.46 | (Gao *et al.*, 2010) |
| 8 | Beichaunghe, Datong | 2.8 | 508 | *Populus bolleana+* Hippophae rhamnoides | 20 | 3.47 | (Gao *et al.*, 2010) |
| 9 | Yangjiawan, Datong | 0.4 | 517 | *Betula platyphylla* | 10 | 3.98 | (Lv *et al.*, 2003) |
| 10 | Yangjiawan, Datong | 0.4 | 517 | *Betula platyphylla+* Hippophae rhamnoides | 23 | 5.44 | (Lv *et al.*, 2003) |
| 11 | Sunan, Zhangye(Gansu) | 0.8 | 402 | *Picea crassifolia* | 155~204 | 2.36 | (Jing *et al.*, 2011) |
| 12 | Sunan, Zhangye(Gansu) | 0.8 | 402 | Sabina przewalskii | 146~226 | 2.38 | (Jing *et al.*, 2011) |

Δ is mean annual temperature (℃),

+ is mean annual precipitation (mm),

*NPP is calculated using the biomass by 0.45 (C content)

Data Source:

Gao GX, Li DQ, Jia JM, Hu WZ, Liu GQ (2007) Research on soil fertility of different species arrangement models in converted farmland land. *Journal of Arid Land Resources and Environment,* **21**, 104-107.

Lv F (2007) The characteristics of habitat succession and its evaluation of different vegetations in datong county. Ph.D Thesis, Beijing Forestry University. Beijing.

Jing WM, Liu XD, Zhao WJ, Ma J (2011) Study On biomass and net prod uctivity of typical forest stand in the Qilian Mountains. *Journal of Gansu Gricultural University,* **6**, 81-85.
